# Crystal structure of a new hybrid compound based on an iodido­plumbate(II) anionic motif

**DOI:** 10.1107/S2056989015023786

**Published:** 2016-01-01

**Authors:** Oualid Mokhnache, Habib Boughzala

**Affiliations:** a Laboratoire de Matériaux et Cristallochimie, Faculté des Sciences de Tunis, Université de Tunis El Manar, 2092 Manar II Tunis, Tunisia

**Keywords:** crystal structure, organic–inorganic hybrid, iodido­plumbate(II), piperazine, 1-D hybrid compound

## Abstract

Crystals of the one-dimensional organic–inorganic lead iodide-based compound *catena*-poly[bis­(piperazine-1,4-diium) [[tetra­iodido­plumbate(II)]-μ-iodido] iodide monohydrate], (C_4_N_2_H_12_)_2_[PbI_5_]I·H_2_O, were obtained by slow evaporation at room temperature of a solution containing lead iodide and piperazine in a 1:2 molar ratio. Inorganic lead iodide chains, organic (C_4_N_2_H_12_)^2+^ cations, water mol­ecules of crystallization and isolated I^−^ anions are connected through N—H⋯·I, N—H⋯O*W* and O*W*—H⋯I hydrogen-bond inter­actions. Zigzag chains of corner-sharing [PbI_6_]^4−^ octa­hedra with composition [PbI_4/1_I_2/2_]^3−^ running parallel to the *a* axis are present in the structure packing.

## Abstract 2

The inorganic part of the crystal structure of the 1-D hybrid compound (C_4_N_2_H_12_)_2_[PbI_5_]I·H_2_O contains corner-sharing [PbI_6_]^4−^ octa­hedra running as zigzag chains along the *a* axis. The organic (piprazineH_2_)^2+^ cations are lodged around the anionic framework. Water mol­ecules and isolated iodine ions play an important role in the structure connectivity.

## Chemical context   

Organic–inorganic hybrid materials offer the opportunity to combine the desirable properties of the organic moiety such as processability, toughness and impact strength with the typical properties of the inorganic part such as high temperature stability and durability. The opto-electronic characteristics of hybrid materials are closely related to the metal cluster size. In recent years, a significant number of organic–inorganic hybrid materials based on lead halide units have been prepared and studied (Billing & Lemmerer, 2006 ▸; Rayner & Billing, 2010 ▸), in particular with self-organized low-dimensional families of lead iodide-based crystals where the [PbI_6_] octa­hedra form one-, two- or three-dimensional networks (Elleuch *et al.*, 2007 ▸; Trigui *et al.*, 2011 ▸). In one-dimensional lead halide hybrid compounds, the inorganic chains may be formed by one, two or three bridging halides, referred to as corner-, edge- and face-sharing polyhedra, respectively. Thanks to their anti­cipated electroluminescence, photoluminescence and non-linear optical properties, these compounds are the most desired ones (Lemmerer & Billing, 2006 ▸). Lead iodide-based hybrid materials are studied extensively for their excitonic and magneto-optical properties. In this work we report the synthesis and crystal structure determination of a new lead iodide hybrid, (C_4_N_2_H_12_)_2_[PbI_5_]·I·H_2_O, (I).
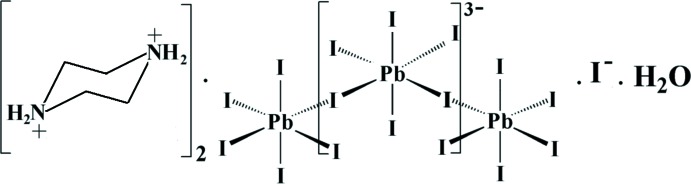


## Structural commentary   

**Figure 1:**
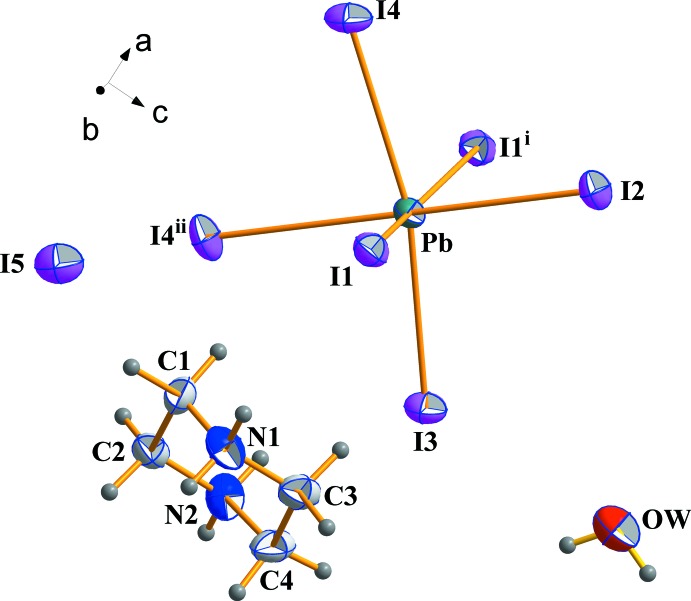
Structural units of the title compound, showing the atom-numbering scheme. Atomic displacement ellipsoids are drawn at the 50% probability level and H atoms are shown as small spheres of arbitrary radius. [Symmetry codes: (i) *x*, 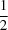 − *y*, *z*; (ii) −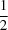 + *x*, 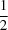 − *y*, 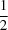 − *z*.]

The structural units of (I) consist of one piperazine mol­ecule, one water mol­ecule, one isolated iodine and one [PbI_6_] unit (Fig. 1 ▸). The electrical neutrality is ensured by two organic mol­ecules of doubly protonated piperazine. 

**Figure 2:**
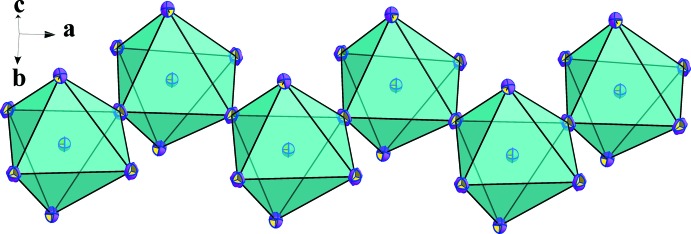
The [PbI_4/1_I_2/2_]^3−^ chain of (I) running parallel to the *a*-axis direction and exhibiting a zigzag conformation.

**Figure 3:**
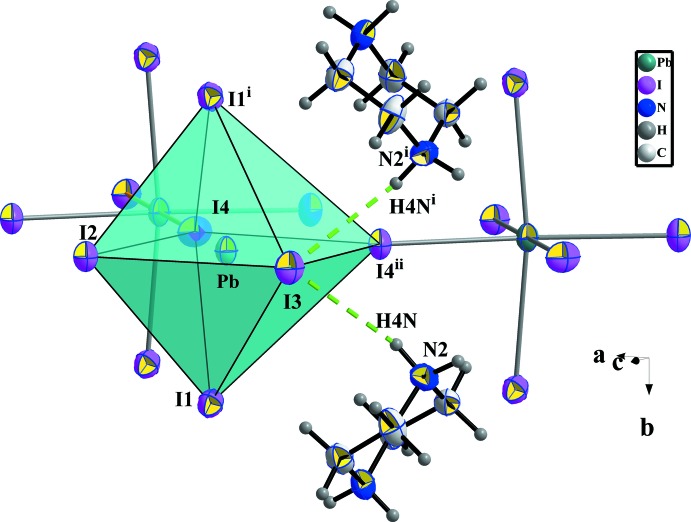
Linkage around one [PbI_6_] octa­hedron formed by two similar octa­hedra and two protonated piperazine cations. Hydrogen bonds are drawn as dashed green lines. [Symmetry codes: (i) *x*, 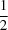 − *y*, *z*; (ii) −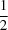 + *x*, 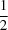 − *y*, 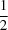 − *z*.]

The main part of the inorganic moiety is composed by the lead Pb^2+^ cation which adopts a distorted octa­hedral coordin­ation. The angles between *cis*-related I^−^ ions range from 85.022 (12) to 96.89 (3)° at most, whereas the *trans* angles deviate from 180° by 12.95 (3)° (Table 1 ▸). Two adjacent corners connect the [PbI_6_] octa­hedron to its neighbours, leading to zigzag chains running parallel to the *a* axis (Fig. 2 ▸). This one-dimensional anionic network leaves empty spaces in which the organic cations are located. The [PbI_6_] octa­hedra establish two strong hydrogen bonds (Table 2 ▸), N2—H4*N*⋯I3 and N2^i^—H4*N* ^i^⋯I3, *via* the I3 corners [symmetry code: (i) *x*, 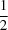 − *y*, *z*] as illustrated in Fig. 3 ▸.

**Figure 4:**
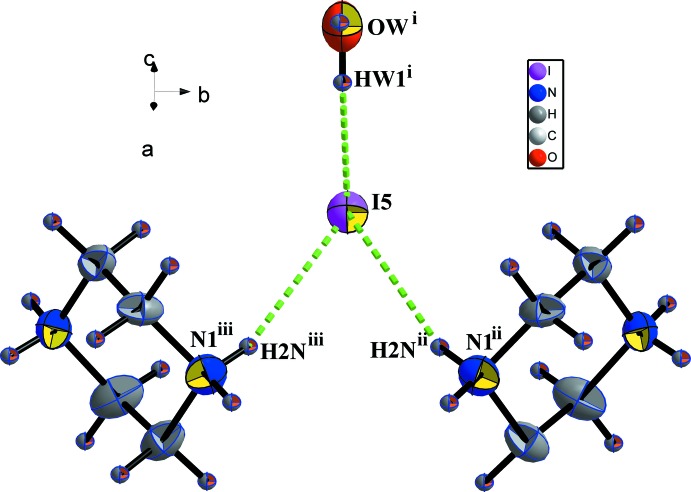
Hydrogen-bonding inter­actions with isolated iodide in (I). [Symmetry codes: (i) 1 − *x*, 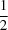 + *y*, 1 − *z*; (ii) 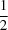 + *x*, 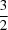 − *y*, 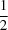 − *z*; (iii) 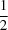 + *x*, *y*, 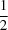 − *z*.]

**Figure 5:**
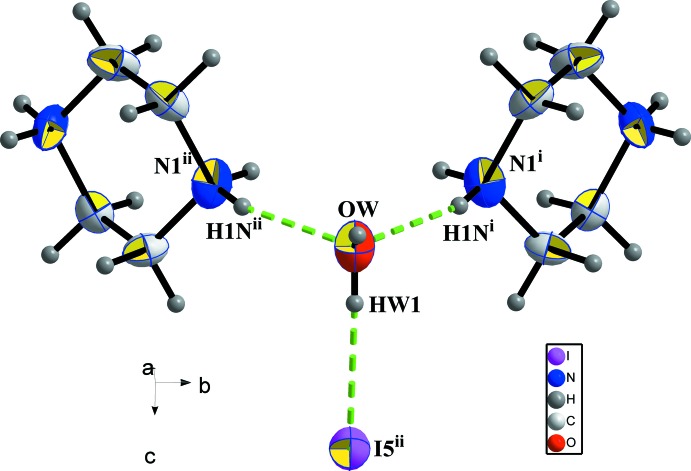
Water mol­ecule hydrogen bonding inter­actions in (I). [Symmetry codes: (i) 1 − *x*, 1 − *y*, 1 − *z*; (ii) 1 − *x*, −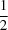 + *y*, 1 − *z*.]

The second part of the inorganic moiety contains a water mol­ecule and the iodide anion I5 linked by a strong hydrogen-bond inter­action (Table 2 ▸). Both are located in the same layers in which the [PbI_6_] octa­hedra are located. As shown in Fig. 4 ▸, the anion I5 is linked to one water mol­ecule by I5⋯H*W*1^i^–O*W* ^i^ [symmetry code: (i) 1 − *x*, 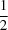 + *y*, 1 − *z*] and two organic cations *via* I5⋯H2*N^i^* ^i^—N1^ii^ and I5⋯H2*N* ^iii^—N1^iii^ [symmetry codes: (ii) 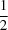 + *x*, 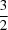 − *y*, 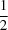 − *z*; (iii) 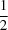 + *x*, *y*, 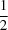 − *z*]. On the other hand, the water mol­ecule is associated to one iodine (I5) *via* O*W*—H*W*1⋯I5^iii^ [symmetry code: (iii) 1 − *x*, −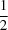 + *y*, 1 − *z*) and to two piperazinium cations *via* O*W*⋯H1*N* ^ii^—N1^ii^ and O*W*⋯H1*N* ^i^—N1^i^ (Fig. 5 ▸). In this configuration, no acceptor was found for H*W*2 and H3*N*.

**Figure 6:**
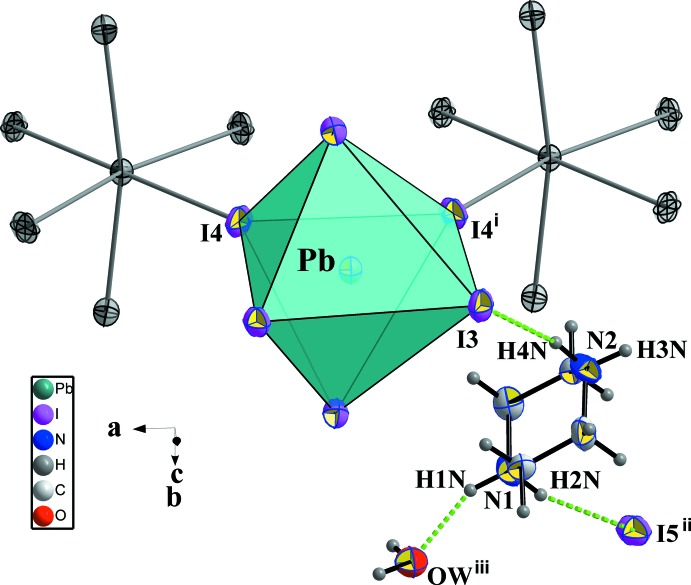
The hydrogen bonding environment of the cation of the title compound. [Symmetry codes: (i) −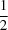 + *x*, 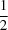 − *y*, 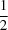 − *z*; (ii) −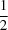 + *x*, 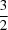 − *y*, 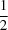 − *z*; (iii) 1 − *x*, 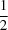 + *y*, 1 − *z*.]

The six-membered piperazinium cation ring adopts a chair conformation. It inter­acts with the inorganic chain *via* strong N2—H4*N*⋯I3 hydrogen bonds with a 2.85 Å bond length (Table 2 ▸ and Fig. 6 ▸). In the crystal structure, the piperazinium cations are also linked to the water mol­ecule by an N1—H1*N*⋯O*W* ^iii^ hydrogen bond and to the iodine anion by N1—H2*N*⋯I5^iii^ hydrogen bonds.

Compared to its homologous hybrids, the structure of the title compound exhibits an original arrangement of the inorganic layers. It is composed by two parts: the first are the [PbI_6_] octa­hedra sharing adjacent corners and so assembling into chains running along the [100] direction. The second original feature is the structural cohesion by water mol­ecules and isolated iodide anions. This structural arrangement will probably have an impact on the dielectric behavior of the material. Luminescence and UV–visible spectroscopy measurements of this compound, coupled to theoritical calculation of the Highest Occupied Mol­ecular Orbital (HOMO) and Lowest Unoccupied Mol­ecular Orbital (LUMO) electronic transitions are in progress.

**Figure 7:**
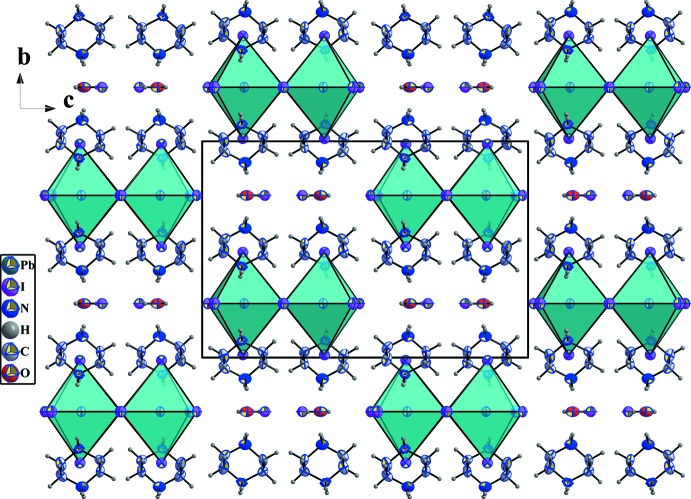
A packing diagram of (I), viewed along the *a* axis showing the alternating organic and inorganic layers. Hydrogen bonds are omitted for clarity.

As shown in Fig. 7 ▸, the structure of (I) is self-assembled into alternating organic and inorganic layers parallel to the *ac* plane. The organic part is made up of (C_4_H_12_N_2_)^2+^ cations located in the voids around the corner-sharing [PbI_6_]^4−^ octa­hedra. The iodine anions and the water mol­ecules connect the organic and inorganic sheets by strong hydrogen-bond inter­actions.

## Database survey   

Using the piperazine-1,4-diium cation scheme in the similarity option of the WEBCSD inter­face (Groom & Allen, 2014 ▸), more than 90 records are found in the CCDC database. Only 24 are inorganic–organic hybrid compounds with several metals Cu, Zn, Co, Bi, Cd, Sb, Au *etc*. The closest chemical composition found is a bis­muth-based compound (II): (C_4_N_2_H_12_)_2_[BiCl_6_]·Cl·H_2_O (Gao *et al.*, 2011 ▸). In spite of the chemical formula similarity, it seems that the ortho­rhom­bic (*Pnma*) title structure is much more regular than the monoclinic (*P*2_1_/*c*) compound (II) with approximately the same cell volume, where the small difference is probably due to the chlorine/iodine substitution. In contrast to the structure of (I), the anionic network in the structure of (II) is 0-D, built up by isolated [BiCl_6_] octa­hedra. The water mol­ecule and the isolated halogen play, in both cases, the same crucial role in the structural cohesion, linking the anionic part to the organic moieties.

## Synthesis and crystallization   

Crystals of the title compound were prepared by slow evaporation at room temperature by mixing 1,4-di­aza­cyclo­hexane (C_4_H_10_N_2_) (2 mol) with a solution of lead iodide PbI_2_ (1 mol) in an equimolar mixture of ethanol and DMF. After several weeks, the obtained crystals were isolated and dried.

## Refinement   

Data collection and structure refinement details are summarized in Table 3 ▸. Hydrogen atoms were placed using geometrical constraints using adequate HFIX instructions (SHELXL) and refined with AFIX instructions. Water hydrogen atoms were found in Fourier difference maps and O—H distances were restrained using DFIX (0.86 Å) and DANG instructions.

## Supplementary Material

Crystal structure: contains datablock(s) I. DOI: 10.1107/S2056989015023786/vn2104sup1.cif

Structure factors: contains datablock(s) I. DOI: 10.1107/S2056989015023786/vn2104Isup2.hkl

CCDC reference: 1429047

Additional supporting information:  crystallographic information; 3D view; checkCIF report

## Figures and Tables

**Table 1:** Selected geometric parameters (Å, °)

Pb—I2	3.0689 (9)	Pb—I4	3.2396 (9)
Pb—I3	3.1511 (9)	Pb—I4^ii^	3.3535 (9)
Pb—I1	3.2173 (8)	I4—Pb^iii^	3.3535 (9)
Pb—I1^i^	3.2173 (8)	O*W*—H*W*2	0.86 (2)
			
I2—Pb—I3	96.06 (3)	I1—Pb—I4	87.185 (13)
I2—Pb—I1	85.021 (12)	I1^i^—Pb—I4	87.185 (13)
I3—Pb—I1	93.943 (13)	I2—Pb—I4^ii^	179.99 (3)
I2—Pb—I1^i^	85.022 (12)	I3—Pb—I4^ii^	83.95 (3)
I3—Pb—I1^i^	93.944 (13)	I1—Pb—I4^ii^	94.978 (12)
I1—Pb—I1^i^	167.89 (2)	I1^i^—Pb—I4^ii^	94.977 (12)
I2—Pb—I4	96.89 (3)	I4—Pb—I4^ii^	83.105 (14)
I3—Pb—I4	167.05 (3)	Pb—I4—Pb^iii^	178.91 (3)

Symmetry codes: (i) 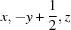; (ii) 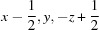; (iii) 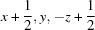.

**Table 2:** Hydrogen-bond geometry (Å, °)

*D*—H⋯*A*	*D*—H	H⋯*A*	*D*⋯*A*	*D*—H⋯*A*
N1—H1*N*⋯O*W* ^iv^	0.90	2.05	2.874 (5)	155
N1—H2*N*⋯I5^v^	0.90	2.69	3.543 (4)	160
N2—H4*N*⋯I3	0.90	2.85	3.656 (4)	151
O*W*—H*W*1⋯I5^vi^	0.86	2.74	3.477 (5)	145

Symmetry codes: (iv) 
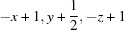
; (v) 
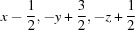
; (vi) 
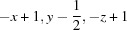
.

**Table 3:** Experimental details

Crystal data	
Chemical formula	(C_4_H_12_N_2_)_2_[PbI_5_]I·H_2_O
*M*_r_	1162.92
Crystal system, space group	Orthorhombic, *P* *n* *m* *a*
Temperature (K)	298
*a*, *b*, *c* (Å)	8.7477 (10), 13.488 (2), 20.336 (3)
*V* (Å^3^)	2399.4 (6)
*Z*	4
Radiation type	Mo *K*α
μ (mm^−1^)	14.75
Crystal size (mm)	0.45 × 0.14 × 0.10

Data collection	
Diffractometer	Enfar–Nonius CAD-4
Absorption correction	ψ scan (North *et al.*, 1968 ▸)
*T*_min_, *T*_max_	0.622, 0.999
No. of measured, independent and observed [*I* > 2σ(*I*)] reflections	3601, 2729, 1941
*R*_int_	0.034
(sin θ/λ)_max_ (Å^−1^)	0.638

Refinement	
*R*[*F* ^2^ > 2σ(*F* ^2^)], *wR*(*F* ^2^), *S*	0.037, 0.086, 1.05
No. of reflections	2729
No. of parameters	105
No. of restraints	3
H-atom treatment	H atoms treated by a mixture of independent and constrained refinement
Δρ_max_, Δρ_min_ (e Å^−3^)	2.00, −1.28

Computer programs: *CAD-4 EXPRESS* (Enraf–Nonius, 1994 ▸), *XCAD4* (Harms & Wocadlo, 1995 ▸), *SHELXS97* (Sheldrick, 2008 ▸), *SHELXL2014* (Sheldrick, 2015 ▸), *DIAMOND* (Brandenburg, 2006 ▸) and *publCIF* (Westrip, 2010 ▸).

## supplementary crystallographic information

### Crystal data

**Table:**

(C_4_H_12_N_2_)_2_[PbI_5_]I·H_2_O	*D*_x_ = 3.219 Mg m^−^^3^
*M**_r_* = 1162.92	Mo *K*α radiation, λ = 0.71073 Å
Orthorhombic, *P**n**m**a*	Cell parameters from 25 reflections
*a* = 8.7477 (10) Å	θ = 13.7–14.7°
*b* = 13.488 (2) Å	µ = 14.75 mm^−^^1^
*c* = 20.336 (3) Å	*T* = 298 K
*V* = 2399.4 (6) Å^3^	Prism, yellow
*Z* = 4	0.45 × 0.14 × 0.10 mm
*F*(000) = 2040	

### Data collection

**Table:**

Enfar–Nonius CAD-4 diffractometer	*R*_int_ = 0.034
Radiation source: fine-focus sealed tube	θ_max_ = 27.0°, θ_min_ = 2.0°
ω/2τ scans	*h* = −11→2
Absorption correction: ψ scan (North *et al.*, 1968)	*k* = −1→17
*T*_min_ = 0.622, *T*_max_ = 0.999	*l* = −1→25
3601 measured reflections	2 standard reflections every 120 min
2729 independent reflections	intensity decay: −1%
1941 reflections with *I* > 2σ(*I*)	

### Refinement

**Table:**

Refinement on *F*^2^	Primary atom site location: heavy-atom method
Least-squares matrix: full	Secondary atom site location: difference Fourier map
*R*[*F*^2^ > 2σ(*F*^2^)] = 0.037	Hydrogen site location: inferred from neighbouring sites
*wR*(*F*^2^) = 0.086	H atoms treated by a mixture of independent and constrained refinement
*S* = 1.05	*w* = 1/[σ^2^(*F*_o_^2^) + (0.0358*P*)^2^ + 1.6589*P*] where *P* = (*F*_o_^2^ + 2*F*_c_^2^)/3
2729 reflections	(Δ/σ)_max_ < 0.001
105 parameters	Δρ_max_ = 2.00 e Å^−^^3^
3 restraints	Δρ_min_ = −1.28 e Å^−^^3^

### Special details

**Table:**

Experimental. Number of psi-scan sets used was 4 Theta correction was applied. Averaged transmission function was used. No Fourier smoothing was applied.
Geometry. All e.s.d.'s (except the e.s.d. in the dihedral angle between two l.s. planes) are estimated using the full covariance matrix. The cell e.s.d.'s are taken into account individually in the estimation of e.s.d.'s in distances, angles and torsion angles; correlations between e.s.d.'s in cell parameters are only used when they are defined by crystal symmetry. An approximate (isotropic) treatment of cell e.s.d.'s is used for estimating e.s.d.'s involving l.s. planes.
Refinement. Refinement of *F*^2^ against ALL reflections. The weighted *R*-factor *wR* and goodness of fit *S* are based on *F*^2^, conventional *R*-factors *R* are based on *F*, with *F* set to zero for negative *F*^2^. The threshold expression of *F*^2^ > σ(*F*^2^) is used only for calculating *R*-factors(gt) *etc*. and is not relevant to the choice of reflections for refinement. *R*-factors based on *F*^2^ are statistically about twice as large as those based on *F*, and *R*- factors based on ALL data will be even larger.

### Fractional atomic coordinates and isotropic or equivalent isotropic displacement parameters (Å^2^)

**Table:**

	*x*	*y*	*z*	*U*_iso_*/*U*_eq_	
Pb	0.70782 (5)	0.2500	0.37128 (2)	0.02909 (13)	
I1	0.74558 (6)	0.48720 (5)	0.37511 (3)	0.03537 (16)	
I2	0.94306 (9)	0.2500	0.48324 (4)	0.0367 (2)	
I3	0.41658 (9)	0.2500	0.46247 (4)	0.0410 (2)	
I4	0.95081 (10)	0.2500	0.25107 (4)	0.0421 (2)	
I5	0.54190 (11)	0.7500	0.19255 (5)	0.0489 (3)	
N1	0.3401 (9)	0.5979 (6)	0.3685 (3)	0.0430 (19)	
H1N	0.2673	0.6433	0.3635	0.052*	
H2N	0.4301	0.6287	0.3680	0.052*	
N2	0.1611 (9)	0.4232 (6)	0.3784 (3)	0.0407 (19)	
H3N	0.0691	0.3951	0.3792	0.049*	
H4N	0.2305	0.3754	0.3828	0.049*	
C1	0.3329 (10)	0.5257 (8)	0.3131 (4)	0.039 (2)	
H1A	0.3455	0.5604	0.2716	0.046*	
H1B	0.4151	0.4779	0.3172	0.046*	
C2	0.1836 (9)	0.4737 (7)	0.3138 (4)	0.035 (2)	
H2A	0.1805	0.4252	0.2787	0.042*	
H2B	0.1019	0.5210	0.3066	0.042*	
C3	0.3199 (10)	0.5469 (8)	0.4320 (4)	0.041 (2)	
H3A	0.4040	0.5012	0.4389	0.050*	
H3B	0.3214	0.5953	0.4672	0.050*	
C4	0.1744 (10)	0.4919 (8)	0.4337 (4)	0.046 (3)	
H4A	0.1677	0.4552	0.4746	0.055*	
H4B	0.0900	0.5386	0.4325	0.055*	
OW	0.4301 (11)	0.2500	0.6369 (5)	0.051 (3)	
HW1	0.393 (13)	0.2500	0.676 (2)	0.080*	
HW2	0.352 (9)	0.2500	0.612 (4)	0.059*	

### Atomic displacement parameters (Å^2^)

**Table:**

	*U*^11^	*U*^22^	*U*^33^	*U*^12^	*U*^13^	*U*^23^
Pb	0.0224 (2)	0.0364 (3)	0.0284 (2)	0.000	0.00002 (19)	0.000
I1	0.0287 (3)	0.0386 (4)	0.0388 (3)	0.0019 (2)	−0.0011 (2)	0.0008 (3)
I2	0.0322 (4)	0.0408 (6)	0.0372 (4)	0.000	−0.0079 (4)	0.000
I3	0.0296 (4)	0.0498 (6)	0.0435 (4)	0.000	0.0088 (4)	0.000
I4	0.0353 (4)	0.0420 (5)	0.0491 (5)	0.000	0.0180 (4)	0.000
I5	0.0525 (5)	0.0392 (6)	0.0550 (5)	0.000	0.0114 (5)	0.000
N1	0.030 (4)	0.042 (5)	0.058 (5)	−0.007 (4)	−0.009 (4)	0.002 (4)
N2	0.044 (4)	0.032 (4)	0.046 (4)	−0.009 (4)	−0.008 (4)	0.006 (4)
C1	0.039 (5)	0.051 (6)	0.025 (4)	−0.002 (5)	−0.001 (4)	0.005 (4)
C2	0.031 (4)	0.035 (5)	0.040 (5)	0.002 (4)	−0.004 (4)	−0.005 (4)
C3	0.035 (5)	0.046 (6)	0.043 (5)	0.000 (5)	0.001 (4)	−0.015 (5)
C4	0.029 (5)	0.067 (8)	0.041 (5)	−0.009 (5)	0.008 (4)	−0.012 (5)
OW	0.046 (6)	0.038 (6)	0.068 (6)	0.000	−0.003 (5)	0.000

### Geometric parameters (Å, º)

**Table:**

Pb—I2	3.0689 (9)	N2—H4N	0.8900
Pb—I3	3.1511 (9)	C1—C2	1.483 (11)
Pb—I1	3.2173 (8)	C1—H1A	0.9700
Pb—I1^i^	3.2173 (8)	C1—H1B	0.9700
Pb—I4	3.2396 (9)	C2—H2A	0.9700
Pb—I4^ii^	3.3535 (9)	C2—H2B	0.9700
I4—Pb^iii^	3.3535 (9)	C3—C4	1.473 (12)
N1—C1	1.490 (11)	C3—H3A	0.9700
N1—C3	1.474 (11)	C3—H3B	0.9700
N1—H1N	0.8900	C4—H4A	0.9700
N1—H2N	0.8900	C4—H4B	0.9700
N2—C4	1.462 (11)	OW—HW1	0.86 (2)
N2—C2	1.493 (10)	OW—HW2	0.86 (2)
N2—H3N	0.8900		
			
I2—Pb—I3	96.06 (3)	H3N—N2—H4N	107.9
I2—Pb—I1	85.021 (12)	C2—C1—N1	109.8 (7)
I3—Pb—I1	93.943 (13)	C2—C1—H1A	109.7
I2—Pb—I1^i^	85.022 (12)	N1—C1—H1A	109.7
I3—Pb—I1^i^	93.944 (13)	C2—C1—H1B	109.7
I1—Pb—I1^i^	167.89 (2)	N1—C1—H1B	109.7
I2—Pb—I4	96.89 (3)	H1A—C1—H1B	108.2
I3—Pb—I4	167.05 (3)	C1—C2—N2	110.0 (7)
I1—Pb—I4	87.185 (13)	C1—C2—H2A	109.7
I1^i^—Pb—I4	87.185 (13)	N2—C2—H2A	109.7
I2—Pb—I4^ii^	179.99 (3)	C1—C2—H2B	109.7
I3—Pb—I4^ii^	83.95 (3)	N2—C2—H2B	109.7
I1—Pb—I4^ii^	94.978 (12)	H2A—C2—H2B	108.2
I1^i^—Pb—I4^ii^	94.977 (12)	C4—C3—N1	111.1 (7)
I4—Pb—I4^ii^	83.105 (14)	C4—C3—H3A	109.4
Pb—I4—Pb^iii^	178.91 (3)	N1—C3—H3A	109.4
C1—N1—C3	110.7 (7)	C4—C3—H3B	109.4
C1—N1—H1N	109.5	N1—C3—H3B	109.4
C3—N1—H1N	109.5	H3A—C3—H3B	108.0
C1—N1—H2N	109.5	N2—C4—C3	111.6 (7)
C3—N1—H2N	109.5	N2—C4—H4A	109.3
H1N—N1—H2N	108.1	C3—C4—H4A	109.3
C4—N2—C2	112.1 (7)	N2—C4—H4B	109.3
C4—N2—H3N	109.2	C3—C4—H4B	109.3
C2—N2—H3N	109.2	H4A—C4—H4B	108.0
C4—N2—H4N	109.2	HW1—OW—HW2	104 (3)
C2—N2—H4N	109.2		

Symmetry codes: (i) *x*, −*y*+1/2, *z*; (ii) *x*−1/2, *y*, −*z*+1/2; (iii) *x*+1/2, *y*, −*z*+1/2.

### Hydrogen-bond geometry (Å, º)

**Table:**

*D*—H···*A*	*D*—H	H···*A*	*D*···*A*	*D*—H···*A*
N1—H1*N*···O*W*^iv^	0.90	2.05	2.874 (5)	155
N1—H2*N*···I5^v^	0.90	2.69	3.543 (4)	160
N2—H4*N*···I3	0.90	2.85	3.656 (4)	151
O*W*—H*W*1···I5^vi^	0.86	2.74	3.477 (5)	145

Symmetry codes: (iv) −*x*+1, *y*+1/2, −*z*+1; (v) *x*−1/2, −*y*+3/2, −*z*+1/2; (vi) −*x*+1, *y*−1/2, −*z*+1.
